# Retraction Note: Microwave radiation-induced grafting of 2-methacryloyloxyethyl trimethyl ammonium chloride onto lentil extract (LE-g-DMC) as an emerging high-performance plant-based grafted coagulant

**DOI:** 10.1038/s41598-026-43305-1

**Published:** 2026-03-10

**Authors:** Siong Chin Chua, Fai Kait Chong, Muhammad Raza Ul Mustafa, Shamsul Rahman Mohamed Kutty, Wawan Sujarwo, Marlinda Abdul Malek, Pau Loke Show, Yeek Chia Ho

**Affiliations:** 1 Civil and Environmental Engineering Department, Universiti Teknologi PETRONAS, 32610 Seri Iskandar, Perak Darul Ridzuan Malaysia; 2 Centre of Urban Resource Sustainability, Institute of Self-Sustainable Building, Universiti Teknologi PETRONAS, 32610 Seri Iskandar, Perak Darul Ridzuan Malaysia; 3 Institute of Sustainable Energy (ISE), Universiti Tenaga Nasional, 43000 Kajang, Selangor Malaysia; 4 Fundamental and Applied Sciences Department, Universiti Teknologi PETRONAS, 32610 Seri Iskandar, Perak Darul Ridzuan Malaysia; 5 Cibodas Botanical Gardens, Indonesian Institute of Sciences (LIPI), Cianjur, West Java Indonesia; 6 Department of Chemical and Environmental Engineering, Faculty of Engineering, University of Nottingham Malaysia, Jalan Broga, Semenyih Selangor Darul Ehsan Malaysia

Retraction of: *Scientific Reports* 10.1038/s41598-020-60119-x, published online 03 March 2020

The Editors have retracted this Article.

After publication, concerns were raised that the brand of the SEM used in the Article is stated to be Perkin Elmer. However, in Fig. 7, the images list Zeiss as the SEM brand. The Authors explained that they did FTIR with a Perkin Elmer machine and SEM with a Zeiss microscope, but the data provided by the Authors was not sufficient to confirm this. Additionally, the Editors requested other data underlying the Article, but the data provided by the Authors did only partially contain metadata that would allow to validate it.

The Editors have therefore lost confidence in the reliability of this Article.

Yeek Chia Ho disagrees with the retraction. Siong Chin Chua, Fai Kait Chong, Muhammad Raza Ul Mustafa, Shamsul Rahman Mohamed Kutty, and Wawan Sujarwo did not respond to the correspondence from the Editors about this retraction. The Editors were not able to confirm the current contact details for Marlinda Abdul Malek and Pau Loke Show.

